# STASISM: A Versatile Serious Gaming Multi-Sensor Platform for Personalized Telerehabilitation and Telemonitoring

**DOI:** 10.3390/s24020351

**Published:** 2024-01-06

**Authors:** Anna Kushnir, Oleh Kachmar, Bruno Bonnechère

**Affiliations:** 1 Elita Rehabilitation Center, 79000 Lviv, Ukraine; okachmar@ic.reha.lviv.ua; 2 REVAL Rehabilitation Research Center, Faculty of Rehabilitation Sciences, Hasselt University, 3590 Diepenbeek, Belgium; bruno.bonnechere@uhasselt.be; 3 Technology-Supported and Data-Driven Rehabilitation, Data Sciences Institute, Hasselt University, 3590 Diepenbeek, Belgium; 4 Department of PXL-Healthcare, PXL University of Applied Sciences and Arts, 3500 Hasselt, Belgium

**Keywords:** multiple sensors, telerehabilitation, assessment, personalized treatment, rehabilomics

## Abstract

Telemonitoring and telerehabilitation have shown promise in delivering individualized healthcare remotely. We introduce STASISM, a sensor-based telerehabilitation and telemonitoring system, in this work. This platform has been created to facilitate individualized telerehabilitation and telemonitoring for those who need rehabilitation or ongoing monitoring. To gather and analyze pertinent and validated physiological, kinematic, and environmental data, the system combines a variety of sensors and data analytic methodologies. The platform facilitates customized rehabilitation activities based on individual needs, allows for the remote monitoring of a patient’s progress, and offers real-time feedback. To protect the security of patient data and to safeguard patient privacy, STASISM also provides secure data transmission and storage. The platform has the potential to significantly improve the accessibility and efficacy of telerehabilitation and telemonitoring programs, enhancing patients’ quality of life and allowing healthcare professionals to provide individualized care outside of traditional clinical settings.

## 1. Introduction

With the increasing need for rehabilitation services worldwide, there is a growing demand to address the challenges associated with traditional rehabilitation approaches [1]. Factors such as a lack of motivation, limited access to healthcare professionals, and the disruption caused by the COVID-19 pandemic have further highlighted the importance of innovative solutions in the field of rehabilitation [2,3,4]. Telerehabilitation, enabled by advancements in technology, has emerged as a promising approach to bridge the gap between patients and healthcare providers, offering convenience, accessibility, and personalized care [5,6].

Traditional rehabilitation programs often face obstacles related to patient motivation and the availability of healthcare professionals. Many patients struggle to maintain consistent motivation throughout their rehabilitation journey, leading to suboptimal outcomes [7,8]. Additionally, the shortage of qualified rehabilitation professionals in certain regions restricts access to specialized care, further exacerbating the challenges faced by patients seeking rehabilitation services [9,10,11]. Furthermore, the COVID-19 pandemic has significantly disrupted the provision of in-person healthcare services, including rehabilitation programs. Lockdown measures, travel restrictions, and concerns about virus transmission have limited patients’ ability to attend regular rehabilitation sessions [4]. All these points highlighted the need to develop innovative solutions to increase the quality and the quantity of rehabilitation exercises patients can perform, either supervised (with clinicians) or unsupervised (e.g., telerehabilitation [12]).

In recent years, commercial games and gamified applications have gained popularity as tools for rehabilitation and therapy [13,14]. These interactive platforms offer engaging and enjoyable experiences for patients, motivating them to actively participate in their rehabilitation programs. By incorporating elements of gamification, telerehabilitation platforms can enhance patient engagement and adherence to prescribed exercises, leading to improved outcomes [15,16]. Serious games can address cultural and contextual factors by offering localized content and incorporating culturally relevant narratives and characters [17]. Cultural sensitivity is crucial in healthcare, and serious games can be designed to resonate with the values, beliefs, and practices of the target communities [18].

The development of the telerehabilitation platform is not new, and recommendations have been made based on experts’ and patients’ opinions. Both highlight the importance of clarity in exercise instructions, ongoing assessments of progress, and constructive feedback. In home environments, patients express feelings of insecurity and apprehension about potential difficulties in execution and the scarcity of detailed exercise instructions in the absence of a physiotherapist. Simultaneously, physiotherapists encounter challenges in overseeing patients’ adherence to home exercise programs [19].

Therefore, the success of telerehabilitation programs relies heavily on appropriate configuration and the inclusion of specific exercises tailored to individual patient needs. Personalization plays a pivotal role in optimizing rehabilitation outcomes, considering patients’ unique conditions, abilities, and goals [20]. A comprehensive understanding of patient characteristics and ongoing progress is essential for designing effective rehabilitation interventions [21]. Another important point that has been highlighted is the training of professionals and students [22]. Indeed, while the overall acceptability of this technology is favorable, its effectiveness could be enhanced through the implementation of an easily accessible user interface, supplemental rehabilitation materials, and continuous training. Additionally, providing timely technical support to therapists would further contribute to the optimization of this technology [23].

The evolution of technology in the field of rehabilitation, in this case, telerehabilitation, is not a recent phenomenon. Nevertheless, in recent decades, technology has undergone significant advancements, unveiling exciting prospects for patient management and assessment. Rehabilitation now benefits from an array of existing technologies, including robotics, muscle and brain stimulation, sensor-based exergames, and virtual reality applications [24,25]. The appeal of technology-supported rehabilitation lies in its ability to provide objective, automated, and, if necessary, blinded assessments, resulting in time saving. It enables the quantifiable evaluation of motor function by considering patient-specific characteristics, including kinematics, activity level, intensity, muscle activity, co-contraction, posture, smoothness of motion during rehabilitation exercises, heart rate, stress levels, and more [26].

Rehabilomics, a holistic approach combining rehabilitation and omics technologies (such as genomics, proteomics, and metabolomics), offers valuable insights into personalized rehabilitation strategies [27,28]. By integrating clinical data, patient profiles, and molecular information, rehabilomics can guide the selection of specific exercises and interventions tailored to individual patients, maximizing their rehabilitation potential.

In this paper, we present a versatile multi-sensor platform designed to address the challenges associated with traditional rehabilitation methods. STASISM combines the benefits of telerehabilitation, gamification, personalized configuration, and rehabilomics to deliver effective and engaging rehabilitation programs. Through the integration of advanced sensor technologies and data analysis techniques, STASISM aims to enhance patient motivation, accessibility to healthcare professionals, and overall rehabilitation outcomes.

The subsequent sections of this paper will delve into the architecture, sensor technologies, data analysis methods, telerehabilitation and telemonitoring applications, and security and privacy considerations, as well as case studies and future directions of STASISM. By leveraging the power of technology and personalized approaches, STASISM strives to revolutionize the field of rehabilitation and improve the quality of life for individuals in need of rehabilitative care.

## 2. Development and Validation of the Platform

The STASISM platform is the result of a co-creation process, where a diverse consortium of experts came together for EU research projects to design an innovative tool for children with motor disabilities. Here, we will briefly describe the collaborative efforts of therapists, medical doctors, software engineers, and data analysis specialists in the platform’s development and its subsequent refinement through real-world user feedback.

### 2.1. A Multidisciplinary Consortium

The development of the platform commenced with the convergence of talents from various disciplines. The collaboration of therapists, medical doctors, software engineers, and data analysis specialists formed the foundation for a rich co-creation process. The platform exemplifies the transformative potential of collaborative co-creation. By uniting experts from different domains and valuing user feedback, the platform’s design and functionality have evolved to benefit those it serves. Embracing the spirit of co-creation, the platform stands as an inspiring model for future interdisciplinary projects seeking to make a positive impact on society.

### 2.2. Piloting across Diverse Contexts

The testing and piloting of the platform involved seven pilot centers situated in different regions across Spain, Ireland, and Ukraine. This strategic selection of diverse locations offered an invaluable array of cultural and economic backgrounds, which played a significant role in the development and refinement of the platform. This approach was instrumental in ensuring the adaptability and inclusivity of the platform to cater to a wide range of needs.

#### 2.2.1. Cultural Diversity

The testing procedure was made more representative of a wider range of cultural backgrounds by the selection of pilot locations located throughout these three countries. The concepts of healthcare, rehabilitation, and technology are all interpreted in a variety of ways by various cultures. The platform was able to develop its user interfaces, content, and engagement techniques by running trials in a variety of cultural settings. This allowed the platform to better align itself with the preferences and expectations of users who come from a variety of cultural backgrounds. It is essential to have this level of cultural sensitivity in order to make telerehabilitation useful and available to people all around the world.

#### 2.2.2. Economic Diversity

Testing the system in regions with varying economic backgrounds allowed the development team to assess the platform’s feasibility across a spectrum of financial constraints. This experience helped shape pricing models, reimbursement strategies, and resource allocation plans for the platform. It also ensured that the benefits of telerehabilitation could be extended to individuals with lower incomes. Economic factors play a significant role in healthcare accessibility and preferences.

#### 2.2.3. Adaptability and Inclusivity

The integration of varied cultural and economic situations throughout the piloting phase underscored the platform’s ability to adjust and accommodate different circumstances, thus promoting inclusiveness. The aforementioned example demonstrated the platform’s capacity to adapt and satisfy a wide range of requirements, preferences, and limitations. The success of the platform in many contexts serves as evidence of its ability to provide a positive influence on a global level by effectively tackling the distinct obstacles encountered by diverse communities.

### 2.3. Listening to User Feedback

The active participation of physicians, patients, and families in the co-creation process of the platform was crucial, and user feedback played a fundamental role in shaping its development. The platform’s usability, clinical effectiveness, and adherence to stringent healthcare standards were significantly influenced by the useful ideas supplied by clinicians who had extensive expertise in rehabilitation methods. Concurrently, the inclusion of families in the feedback process recognizes their substantial contribution to providing support and encouragement to patients throughout their recovery process. The viewpoints provided by the participants provided a thorough understanding of the ways in which the platform influenced the everyday lives of patients and contributed to the development of features that could improve the overall rehabilitation process. The establishment of a continuous feedback loop throughout this process functioned as a catalyst for enhancing the system. This enabled timely modifications and updates to be made in response to the changing requirements and preferences of healthcare professionals and patients. Consequently, STASISM was able to effectively adapt to the ever-changing landscape of healthcare and rehabilitation.

Thanks to feedback, a thorough approach has been put into effect to optimize the user experience. The user interface is designed to facilitate easy navigation, allowing seamless engagement for individuals with different degrees of technological expertise. The platform’s user interface and functionalities have been enhanced through comprehensive user testing and feedback gathering. The platform undergoes frequent evaluations to identify and address any potential challenges in user experience. Furthermore, users are provided with a user guide and onboarding resources to efficiently acquaint themselves with the system. These stages ensure that the STASISM platform is both technologically robust and user-friendly, meeting the diverse needs of patients and healthcare professionals involved in rehabilitation exercises and assessments.

### 2.4. Ongoing System Enhancement

The feedback and evaluation given by users have been essential in shaping and enhancing the STASISM platform. Through the active participation of clinicians, patients, and their families in the collaborative development process, we gained crucial input regarding the platform’s usability, therapeutic efficacy, and compliance with healthcare standards. The implementation of an ongoing feedback loop throughout the development process served as a catalyst for enhancements, facilitating prompt adjustments in accordance with the evolving needs and preferences of healthcare professionals and patients. The data collected from user interactions, including mobility data, exercise performance, and user preferences, are systematically analyzed to provide evidence-based insights into the effectiveness of the rehabilitation programs. The software leverages user feedback and robust analytics to acquire a comprehensive comprehension of patient progress. This allows the platform to offer customized interventions and refine treatment programs using quantifiable metrics.

## 3. The Platform: STASISM

### 3.1. Hardware

The platform is intentionally designed to operate with readily accessible and affordable equipment, ensuring widespread availability. Users are required to have (i) a PC or laptop running Windows 10 or a later version and, contingent upon the objectives of their rehabilitation program, (ii) an off-the-shelf web camera, (iii) a balance board, and (iv) internet connection. This hardware configuration not only fosters accessibility but also contributes to the platform’s cost-effectiveness. The recommended requirements for optimal use of the system are presented in Table 1.

However, a salient feature of this platform is its adaptability in a landscape characterized by the rapid evolution of technology. It encapsulates an extensive array of motion and balance input control systems that are in a state of refinement and advancement. These encompass various innovative technologies, such as wearable sensors, smart textiles, 3D cameras, and more. Each of these control systems has undergone meticulous engineering to ensure the delivery of highly precise therapeutic outcomes.

At the core of the platform’s functionality is its comprehensive approach, surpassing traditional evaluation methodologies and predefined exercise regimens. This approach includes unstructured free-play sessions, during which the software conducts an exhaustive assessment of a user’s performance. This in-depth analysis reveals nuanced areas of concern and potential for improvement. Subsequently, the platform furnishes users with precise and personalized recommendations tailored to their specific therapeutic goals.

The current system leverages cutting-edge sensor technology and harnesses the power of advanced machine learning algorithms for effective data acquisition, analysis, and interpretation related to an individual’s movement and balance. Utilizing these data, the platform employs evidence-based techniques to discern subtle deficits and identify areas with the potential for improvement in an individual’s physical and cognitive capabilities. This technologically driven approach not only elevates the quality of care but also paves the way for a paradigm shift in the field of rehabilitation, offering tailored, data-driven therapeutic interventions.

### 3.2. Architecture of the Data Collection

The platform currently uses two principal hardware to capture user motion data, thus ensuring a full evaluation of their physical ability. The aforementioned techniques encompass the following.

The balance-board device functions as an instrument for measuring the user’s center of pressure and monitoring their movement along two fundamental axes—the anteroposterior and mediolateral directions.

For motion analysis, the system uses advanced machine learning models to convert webcam video images into accurate 3D positional data. This model is capable of detecting and identifying up to 182 unique landmarks within the camera frame. This methodology enables a comprehensive comprehension of the user’s locomotion within a 3D environment, hence enabling the evaluation of complicated motion patterns and bodily orientations [29,30].

In order to guarantee the highest level of precision, the system employs a sampling frequency of 20 Hz to collect data. The method of collecting high-frequency data creates a comprehensive and finely detailed depiction of the user’s mobility over a period of time.

**Figure 1:**
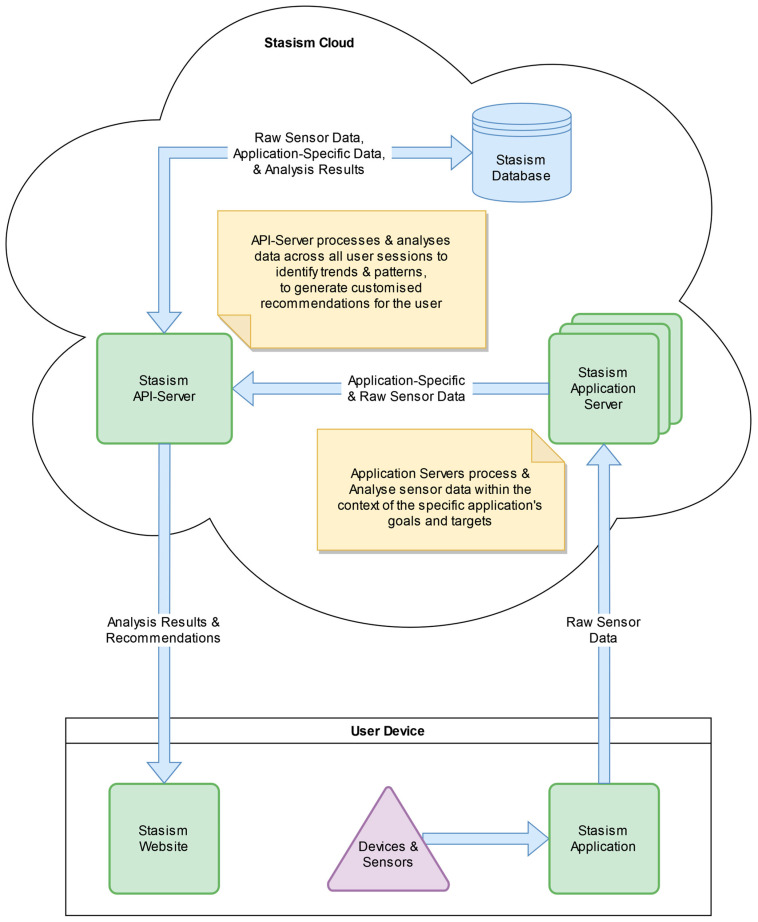
Architecture of the telerehabilitation platform.

After acquiring the unprocessed motion data, the data are translated into program controls, encompassing various operations, such as character movement, item selection within a user interface, and the triggering of special effects. Following this, the data that have been understood are sent to the appropriate application server for additional analysis and secure storage (see Figure 1).

A comprehensive examination is conducted within the application servers. The data undergo thorough examination within the particular context of the application and its operational control mechanisms. This examination produces a set of standardized scores that maintain comparability across various application scenarios. The unprocessed motion data, along with the derived scores, are thereafter sent to the central API server for additional detailed analysis and secure long-term storage.

The API server conducts a comprehensive analysis of the data it receives, calculating a diverse range of outcomes, which may include but are not limited to ranges of motion (RoM), smoothness, symmetry, angular velocity, and acceleration [31].

The computed variables provide significant insights into the user’s physical performance and identify potential areas for improvement.

Additionally, the API server has exceptional proficiency in conducting longitudinal analysis through the examination of user application sessions and the observation of data trends over an extended period. This functionality facilitates the recognition of advancements and, if needed, the indication of domains that might necessitate attention or therapeutic action.

The results of these thorough studies are not only made available for user examination but also serve as the basis for delivering individualized recommendations. The system uses the extensive dataset available to assist users in making informed decisions regarding the selection of applications, control schemes, and difficulty levels that are most suited to their therapeutic requirements. The implementation of this comprehensive strategy guarantees that the platform maintains a leading position in scientific and technical progress aimed at improving physical health and maximizing human capabilities.

### 3.3. Security of Patient Data and Data Privacy

Ensuring the security and confidentiality of patient data is paramount in telerehabilitation platforms. Patients require reassurance regarding the secure handling of their personal health data during telemedicine encounters. To ensure the secure transfer and storage of data, a set of specific measures is diligently followed:

1. The real-world identity of the patient is not stored within our system. The responsibility lies with the clinic to link the STASISM account with the patient’s identity.
2. Rigorous adherence to GDPR standards is maintained, with a dedicated data protection officer (DPO) overseeing the handling of all data.
3. In compliance with EU regulations, all data are stored exclusively within the European Union.
4. Stringent security measures are applied to all traffic to and from the server, ensuring encryption for enhanced data protection.
5. The database itself is encrypted, providing an additional layer of security to safeguard stored information.
6. A robust security protocol is in place, where all logins are fortified with two-factor authentication (2FA), bolstering the integrity of user access and further securing the system against unauthorized entry.

These protocols were demonstrated to the European Commission within the AbleGames projects, a context where our focus on addressing the needs of a vulnerable patient group underscored the importance of robust data security.

### 3.4. Therapeutic Programs

The rehabilitation platform has a wide range of applications, making it suitable for diverse environments such as clinical facilities, community centers, and private residences. Patients have the option to interact with the system based on the instruction provided by a clinician or choose to independently engage with the virtual personal therapist, which is a feature driven by artificial intelligence (AI). The AI component possesses the capability to autonomously propose rehabilitation exercises or serious games, taking into account the patient’s continuous advancement, thereby customizing the experience to suit their individual requirements and skills.

The primary aim of the virtual personal therapist is to function as a supportive companion, regardless of the patient’s previous exercise background. This program guides individuals through a progression from a beginner level to an advanced stage, with a primary emphasis on enhancing exercise technique and optimizing performance while maintaining a high level of motivation. The platform uses ongoing assessments to track the patient’s advancement and adaptively modifies different exercise-specific parameters when the patient demonstrates readiness for more advanced tasks. This process guarantees that the rehabilitation process of each patient is carefully configured and personalized, thereby improving the effectiveness of the platform and facilitating a smooth progression from fundamental exercises to more complex and demanding ones.

### 3.5. (Tele) Rehabilitation Exercises

The platform provides a wide array of rehabilitation exercises that are specifically tailored to cater to individuals with diverse motor abilities and specific therapeutic goals. In order to facilitate patients’ prompt comprehension of exercise expectations, the first phase entails the presentation of video demonstrations for each individual activity. This visual depiction provides patients with a concise and easily comprehensible overview, thereby enhancing their comprehension of essential topics and exercise recommendations.

The system takes an additional stride by including a visual indicator functionality that effectively accentuates the particular anatomical regions focused on throughout each exercise. The platform uses overlays to deliver clear and unambiguous indications, effectively directing attention towards the specific regions of emphasis during the activity. This novel methodology streamlines the process of identifying the specific body areas that necessitate focused attention, hence augmenting patients’ comprehension of correct technique and active involvement.

During physical activities, it provides immediate support to individuals, enabling them to rapidly correct any mistakes. The system actively monitors the patient’s motions and technique during the session, interrupting and providing quick instruction in the event of faults or deviations. The implementation of this proactive strategy guarantees that patients adhere to the correct form and complete the exercises with precision.

Recognizing the inherent difficulty of inundating novice individuals with an abundance of knowledge, the platform employs a progressive methodology to effectively tackle this issue. As the patient advances through each exercise, the platform systematically incorporates supplementary aspects for the patient to concentrate on, thereby maintaining a consistent and feasible rate of learning.

A prevalent obstacle encountered by individuals during rehabilitation exercises is the difficulty of discerning progress across successive sessions. Nevertheless, the system tackles this matter by employing a self-competitive methodology, which allows individuals to observe and experience noticeable improvements in their capabilities as time progresses. STASISM confers patients with a distinct perception of their progress, furnishing concrete proof that functions as a catalyst for motivation, strengthening commitment, and fostering continued engagement in physical treatment.

As an example, to illustrate the whole process, we will use a specific exercise known as the “4-point standing”. In this task, participants are instructed to sustain a weight-bearing position on their hands and knees for a period of 10 s, using a web camera controller. Progress can be assessed by quantifying the accuracy of the exercise, which is determined by measuring the angle between joints, as well as the duration required to execute the activity. Patients are provided with feedback through the process of comparing their current outcomes with their previous performances. As a result, physical therapists have the ability to evaluate the advancement of their patients by employing a quantifiable metric, facilitating accurate monitoring and rehabilitation focused on achieving specific objectives.

### 3.6. Serious Games

The platform includes several games controlled by body, hand, and mouth movements. Each game includes settings to adjust according to the player’s abilities and preferences, including level complexity, image intensity, and features of the game scenario. Games automatically adjust to the player by their physical abilities and skill level. Some games suit children, while others can benefit and interest adult patients. Participation is crucial for people with motor disabilities, and there are multi- and cooperation player modes that allow playing with peers worldwide.

The descriptions of the games, with their main therapeutic exercises, are presented in Table 2.

### 3.7. Third-Party Games

One specificity of this platform is to provide a large number of third-party games that are fully integrated into the platform and can be used for rehabilitation and assessment purposes (see here below). The following games can be played on the platform: Aim Lab, Angry Birds 2, Angry Birds Friends, Beach Buggy Racing 2, Bubble With 3 Saga, Call of Duty: Vanguard, Call of Duty: Modern Warfare II, Candy Crush Saga, Capcom Fighting Collection, Coloring Book & Painting, Cooking Diary, Darkwood, Diablo IV, Doom 64, Draw & Color for Kids, Drawtopia, Filament, Heart Box, Hearthstone, Hill Climb Racing, Katana Fruit, Magic Jigsaw Puzzles, Microsoft Jigsaw, Microsoft Mahjong, Microsoft Solitaire Collection, Microsoft Ultimate Word Games, Minecraft, Moonstuck, Overcooked 1 and 2, Pinball Arcade, Pool Billiard Championship, Roblox, Sky Force Reloaded, Superflight, The Chess Lv.100.

### 3.8. Assessment Tools

Using validated assessment tools is of the utmost importance in the context of rehabilitation platforms. The algorithms used a variety of deep-learning algorithms but mainly R-CNN and YOLO for the image analysis [32]. The accuracy and reliability of these methods are crucial for obtaining meaningful data and ensuring proper evaluation of patients’ progress [33]. By integrating validated assessment tools within the same platform used for rehabilitation, significant time savings can be achieved.

A user-friendly tool has been developed for exploring temporal statistics. The platform offers an overview of statistical trends over time, allowing users to gain insights into temporal patterns. The interface features two primary views: “Active Times” (set as the default) and “Motion”, providing users with distinct perspectives on temporal dynamics. Additionally, the tool allows users to adjust the time period, offering a choice of predefined presets or the option to input custom start and end dates. This flexibility enables researchers to tailor their analysis to specific research questions, enhancing the precision and relevance of the temporal insights derived from the data. Overall, this platform simplifies the exploration of temporal statistics, making it accessible and valuable for researchers in various scientific domains.

This combination streamlines the process, allowing clinicians and therapists to seamlessly switch between rehabilitation exercises and assessments without the need to access separate systems. The synergy between rehabilitation and assessment on a single platform not only enhances efficiency but also fosters a comprehensive approach to patient care, facilitating a deeper understanding of individual needs and tailoring interventions accordingly [34]. This integration empowers healthcare professionals to make well-informed decisions, optimize treatment plans, and ultimately improve patient outcomes.

**Figure 2:**
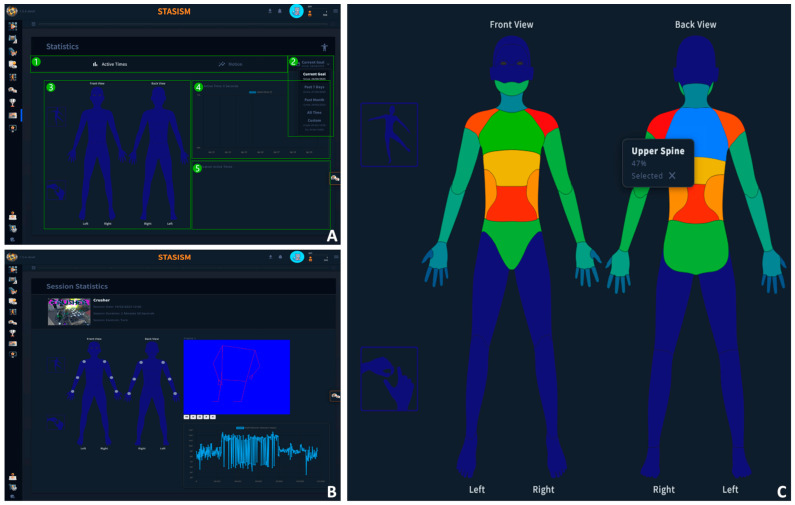
(**A**). Overall representation of the system; (1) tab bar, (2) period selection, (3) heatmap, (4) graph, and (5) application list. (**B**). Statistics session showing data from the selection session and landmark/angle. (**C**). Heatmaps showing the relative time for each body part (based on control types used).

Some functionalities are presented in Figure 2.

#### 3.8.1. Static Balance Test

Designed to assess fundamental stabilometry parameters, this tool determines the center of pressure displacement when an individual maintains a static standing position on the balance board. The tool calculates essential displacement and speed parameters in the anterior–posterior and medial–lateral directions by conducting three ten-second trials using the following previously validated methods [35,36]:

CP anterior–posterior (CPap) and mediolateral (CPml) displacements were obtained from the four strain gauge loads located at the four corners of the WBB using Equations (1) and (2):

(1) CPap=(FR+PR)−(FL+PL)

(2) $$CPml=\left(FL+FR\right)-(PL+PR)$$

where *PL*, *PR*, *FL*, and *FR* are the displacement values from the posterior left, posterior right, front left, and front right WBB sensors, respectively.

Previous works have shown that the time interval between samples of WBB was inconsistent; therefore, linear interpolation of the raw signals of WBB sensors was applied to obtain a regular sample rate.

From those displacements the nine studied parameters were computed using the following equations:

The total displacement of sway (*DOT*) using Equation (3):

(3) $$DOT=\sum_{i=1}^{N}\sqrt{{CPap(i)}^{2}+{CPml(i)}^{2}}$$

The area of the 95% prediction ellipse (often referred to as the 95% confidence ellipse) using Equation (4):

(4) $$Area=\pi \times prod\left(2.4478\times \sqrt{svd\left(eig\left(cov\left(CPap,CPml\right)\right)\right)}\right)$$

The distance between the maximum and minimum COP displacement using Equations (5) and (6):

(5) AP RoM=max(CPap)−min(CPap)

(6) ML RoM=max(CPml)−min(CPml)

The dispersion of COP displacement from the mean position using Equations (7) and (8):

(7) $$AP\;SD=\frac{1}{N}\sqrt{\sum_{i=1}^{N}{CPap(i)}^{2}}$$

(8) $$ML\;SD=\frac{1}{N}\sqrt{\sum_{i=1}^{N}{CPml(i)}^{2}}$$

The mean *AP* and *ML* velocity of COP displacement using Equations (9) and (10):

(9) $$AP\;velocity=\frac{f}{N}\;\sum_{i=1}^{N-1}\left|CPap\left(i+1\right)-CPap\left(i\right)\right|$$

(10) $$ML\;velocity=\frac{f}{N}\;\sum_{i=1}^{N-1}\left|CPml\left(i+1\right)-CPml\left(i\right)\right|$$

The *AP* and *ML* displacements of the total COP sway divided by the total duration of the trial using Equation (11):

(11) $$TMV=\frac{f}{N}\;\sum_{i=1}^{N-1}\sqrt{{\left(CPap\left(i+1\right)-CPap\left(i\right)\right)}^{2}+{\left(CPml\left(i+1\right)-CPml\left(i\right)\right)}^{2}}$$

To ensure accuracy, the results of the static balance assessment tool were validated by comparing them with data obtained through the traditional stabilometry methods.

#### 3.8.2. The Dynamic Balance Test (DBT)

The dynamic balance test serves as a diagnostic tool to assess the capacity of the person to shift weight. During the DBT, the person stands on the balance board and follows on-screen instructions to shift their center of gravity to the left or right. They are required to maintain this position within specific limits for 3 s. As the test progresses, the limits for holding the position become narrower, demanding more precise balancing. The patient’s balancing abilities are characterized by the number of rounds completed within the designated time frame. The displacement of COP is also recorded during all serious games rehabilitation exercises to be later analyzed, using the same parameters as for the static balance, to inform about the dynamic balance control.

To assess dynamic motor control the timed Up and Go can also be recorded as important indicators of lower limb functions [37].

#### 3.8.3. Ranges of Motion

In addition to the aforementioned capabilities, the platform offers a comprehensive evaluation of upper limb ranges of motion (RoM), encompassing critical metrics such as shoulder flexion–extension, shoulder abduction–adduction, shoulder rotations, elbow flexion, and elbow supination. The MediaPipe technique was used to compute the RoM [38]. A key attribute of the MediaPipe technique lies in its ability to mitigate noise in pose prediction or classification through the application of exponential moving averages. This process facilitates the extraction of the closest pose cluster, enabling the calculation of probabilities for each cluster and their utilization in temporal smoothing [39].

These assessments adhere to the established standards and conventions set forth by the International Society of Biomechanics (ISB) [40], ensuring uniformity and adherence to recognized guidelines in reporting results. This approach enables precise and standardized measurement of upper limb mobility, contributing to the platform’s effectiveness in facilitating rehabilitation and tracking progress with the highest degree of accuracy and reliability.

#### 3.8.4. Rapid Measure Tool

The platform provides clinicians with the capacity to create personalized assessments that are tailored to the specific requirements of their patients, thereby enabling them to enhance the quality of their outcomes. The tests described cover a comprehensive set of diagnostic tools that have been carefully devised to evaluate a number of factors, such as balancing function, range of motion, and the accuracy of manual dexterity.

In addition, the platform maintains an archive of historical data, which includes detailed records of full-body movement and other relevant information for each assessment outcome. The data available serve as useful assets for clinicians, providing them with a comprehensive historical background that assists in the development of personalized rehabilitation and the monitoring of patient advancements.

### 3.9. Advance Analytics

All movement data collected during exercises, assessments, and games seamlessly integrate into the patient’s comprehensive data profile within the platform.

Complete 3D analysis of the upper limb motion performed during the rehabilitation exercises can be performed and later analyzed. The methodology has been presented elsewhere [31]. The SPARK algorithm contributes to improved smoothness in the system [41]. To augment the precision of calculations, the introduction of additional constraints can further refine and enhance the algorithm’s performance.

This compilation of movement data serves as a valuable resource for therapists, enabling them to conduct thorough reviews and analyses. By accessing the platform, therapists gain a holistic view of the patient’s overall state and progress, enhancing their ability to make informed decisions and optimize treatment plans.

STATISM offers therapists a robust analysis capability, providing detailed examinations of each exercise. The platform presents exercise-specific calculations and relevant data, facilitating in-depth analysis. This empowers therapists to delve into the information available through the platform, making evidence-based decisions to tailor interventions effectively.

Leveraging exercise-specific calculations and data, therapists gain comprehensive insights into the patient’s performance and progress. They can examine variables such as range of motion, angular velocity, acceleration, rate of change of acceleration, repetitions, duration, and other relevant metrics specific to each exercise. This detailed analysis aids therapists in identifying trends, tracking improvements, and assessing the effectiveness of prescribed interventions.

Note that the calculations remain dynamic and are continually refined to optimize performance. Raw data from sensors are consistently stored at a rate of 20 frames per second, eliminating the need to preselect specific calculations. This approach allows us the flexibility to rerun calculations based on input from therapists, researchers, doctors, and other stakeholders, ensuring that the platform can adapt to the evolving preferences and requirements of its users.

### 3.10. Clinical Validation of the System

We performed a pre-post experimental study with two groups of children with cerebral palsy to assess the effects of the STASISM serious games on balance function. Participants were the patients of the tertiary care center in Ukraine. The study protocol met the criteria of the Helsinki Declaration and received the ethical approval of the local ethical commission. Twenty-five children with cerebral palsy (mean age 11.1 (2.7) years old, 10 girls, mean height 128 (12) cm, mean weight 28.1 (5) kg) were randomly selected for experimental (*n* = 13) or control groups (*n* = 12). All participants had 8–9 sessions of serious game therapy for 15–20 min over a two-week period, representing a total training time of 150–180 min. The experimental group played STASISM games, and the participants from the control group had game sessions with commercial video games using a handheld Wii Remote. Both groups received the same conventional treatment. Outcome measures were a Trunk Control Measurement Scale (TCMS) [42], the Timed Up and Go Test, and the data from the Static Balance Test and the DBT (the embedded diagnostic tests from the platform). After two weeks of training in the experimental group, the score of the TCMS rose by 4.5 ± 3.5 points (*p* < 0.05), and the results of the DBT increased by 0.88 [IQR = 0.88] points (*p* < 0.05); however, these scores did not change substantially in the control group.

The implementation of the system at two rehabilitation institutions in Ukraine not only demonstrates its efficacy but also highlights its potential as a viable solution for addressing the scarcity of healthcare experts in different regions [43]. Ukraine, similar to numerous other regions, encounters the predicament of restricted availability of proficient healthcare professionals owing to a range of circumstances, encompassing geographical limitations, paucity of resources, and economic considerations. The use of the newly developed product in several domains holds the potential to yield substantial impact through the provision of essential rehabilitative assistance, even in situations where a whole cadre of healthcare experts is not available. This exemplifies the platform’s adaptability in addressing crucial healthcare deficiencies, rendering it an essential instrument in guaranteeing that patients residing in disadvantaged areas have the necessary rehabilitative therapy. The effective implementation of STASISM in Ukraine not only underscores its effectiveness but also its capacity to tackle a significant worldwide healthcare issue, namely the shortage of healthcare workers.

### 3.11. Comparisons with Commercial Systems

To ease the visualization of the different functions and compare STASISM with other commercially available (tele)rehabilitation platforms, we performed a quick overview of the existing and most used system. The results are presented in Table 3.

## 4. Discussion

With many benefits over conventional in-person care, telerehabilitation has emerged as a paradigm-shifting strategy in the field of rehabilitation [48]. Patients can access rehabilitation services remotely by using telecommunications technologies, overcoming obstacles like geographic distance and a shortage of medical experts [49]. The presented flexible and multi-sensor platform advances telerehabilitation by using a variety of sensors and data processing methods to deliver tailored and efficient telerehabilitation programs.

The novelty of the presented platform lies in its multi-sensor approach, gamification strategy, and ability to address challenges in traditional rehabilitation, offering a potentially effective solution for improving patient care and outcomes [50].

This platform aligns with current paradigms of patient-centric care [51] and personalized rehabilitation [52]. The platform’s all-encompassing approach aligns with the latest theoretical frameworks in rehabilitation science and expands the discussion on patient-centered approaches [53]. We explore the practical implications of the system on healthcare delivery, patient outcomes, and rehabilitation methods, focusing on its potential for transformation. The scope of our research includes the anticipated advantages of better accessibility, promoting increased patient involvement, and enabling data-driven, customized therapies [54].

A key element of telerehabilitation is telemonitoring, which permits ongoing tracking of patients’ development for both patients and clinicians, gives feedback to the patients assuring them that they have performed the exercises the way they are supposed to do them, and makes prompt treatments possible. With the help of STASISM’s integrated telemonitoring features, healthcare professionals may remotely monitor patients’ progress during rehabilitation, gauge how well they are following instructions, and make required adjustments in real time. The ability to provide ongoing assistance and direction is given to healthcare personnel by this constant follow-up, which improves the efficacy of rehabilitation programs [55]. This system can be coupled with other available technologies, such as mHealth, to perform highly multidimensional telemonitoring and surveillance [56].

Big data analytics have completely changed the healthcare sector [57], while, currently, rehabilitation seems to be a little behind in this area [58]. In this optic, the presented platform uses massive amounts of validated sensor data from numerous sources to collect and have, therefore, the ability to both collect and analyze big data. A deeper understanding of patient development, rehabilitation trends, and therapy effectiveness is made possible by these multiple sources and types of data. STASISM enables evidence-based decision-making by utilizing big data analytics, resulting in more individualized and effective rehabilitation solutions [59,60,61].

The success of rehabilitation results is significantly influenced by personalization. By creating patient profiles that include physiological, kinematic, and environmental data through thorough data gathering and analysis, the platform adjusts rehabilitation plans to fit the needs and peculiarities of each patient. The platform selects and prescribes particular exercises and interventions that are in line with patients’ abilities, preferences, and goals thanks to this tailored approach, which encourages improved involvement and better rehabilitation results [62].

Healthcare professionals can adopt this new paradigm as a useful tool within their current care frameworks thanks to seamless integration. Healthcare providers can acquire a comprehensive understanding of patients’ rehabilitation status and make well-informed decisions about their care by incorporating the results from this platform into electronic health records (EHR) systems and securely sharing pertinent patient data [63]. Through this integration, rehabilitation specialists made sure to play a significant role throughout the patient’s whole healthcare experience, promoting continuity of care and boosting therapeutic results [64].

The adaptability of the system, which combines a variety of sensor technologies and offers a thorough picture of patients’ recovery process, is one of its benefits. The platform’s gamified strategy addresses the issue of lack of motivation frequently connected with conventional rehabilitation approaches by improving patient motivation and adherence to recommended exercises [65]. Additionally, by incorporating rehabilomics principles, rehabilitation programs can be personalized to improve results for specific individuals while offering new possibilities for continuous patient telemonitoring and follow-up [66].

Furthermore, in order to successfully integrate the STASISM platform into various healthcare ecosystems, it is crucial to carefully assess the technological, cultural, and socio-economic risks associated with its development and deployment. Technological risks encompass concerns related to hardware compatibility, the continuous development of sensing technologies, and weaknesses in data security [67]. From a cultural standpoint, issues arise due to differences in user preferences, perceptions of healthcare, and acceptability of technological interventions [65]. Socioeconomic aspects refer to the financial limitations that impact user access to healthcare and the varied infrastructures of healthcare systems [68]. Another essential factor in the successful implementation of digital health solutions is individuals’ familiarity with technology (digital health literacy) and their willingness to embrace digital tools for health-related purposes [69]. The readiness of users to engage with technological platforms plays a pivotal role in the effective adoption and utilization of digital solutions, influencing aspects such as user experience, adherence to prescribed interventions, and overall satisfaction with health technology [70]. Recognizing and mitigating diverse levels of technological familiarity among users is imperative for maximizing the impact and accessibility of digital health solutions in varied populations. A prior study has emphasized the importance of integrating the unique requirements of individuals with disabilities, caregivers, and healthcare professionals right from the design phase of digital healthcare services. This underscores the potential to advance digital health equity by prioritizing inclusivity and addressing specific user needs within the conceptualization and development of technological solutions [71].

A mitigation plan entails periodic revisions to synchronize with advancing technology, the creation of culturally appropriate content, and the continuous integration of user feedback. Technological risks are mitigated by consistently checking and updating hardware requirements and cybersecurity policies. The incorporation of multiple cultural viewpoints in the platform’s design, content, and interaction tactics helps to mitigate cultural hazards. To mitigate socio-economic risks, one must take into account diverse economic backgrounds during testing, ensuring that the approach is cost-effective and refining pricing models. By adopting this comprehensive strategy, STASISM is able to effectively address and minimize potential obstacles in several areas such as technology, culture, and socioeconomics.

For this platform to be widely used, existing healthcare systems must be integrated with it. Collaboration with healthcare providers and seamless interaction with EHR systems provide effective data sharing, faster processes, and increased care coordination [6]. Incorporating STASISM into broader telehealth projects is also made possible by this integration, guaranteeing its compatibility with current medical infrastructure.

The STASISM platform acknowledges particular limitations that necessitate meticulous examination. A crucial aspect to take into account is the dependence on internet connectivity for accessing the platform while recognizing the challenges that may develop in regions with limited connectivity [72]. Efforts are currently underway to enhance accessibility and ensure use across a diverse spectrum of technological proficiencies. Moreover, much focus has been dedicated to resolving concerns around data security and privacy. The platform employs robust security features, including encryption techniques and stringent access restrictions, to safeguard patient data. Regular audits and evaluations are conducted to ensure the highest degree of data security. Consistently improving the platform requires ongoing engagement in talks with consumers, healthcare professionals, and technological experts. This entails addressing any emerging constraints and ensuring that the platform stays pertinent and efficient across various healthcare environments. A last important limitation is the limited reimbursement of telerehabilitation services in many countries, which constitutes a significant barrier to the widespread adoption of such services [73,74].

To overcome these limitations, there are many potential future improvements and extensions for this system. Emerging sensor technologies, such as wearables and smart home sensors, can be added to the platform to further enhance it and collect more thorough data for an overall picture of patients’ rehabilitation progress. Data analysis capabilities can be improved by further integrating AI and machine learning algorithms, offering real-time insights and predictive analytics. Further, increasing patient participation is the incorporation of virtual reality and augmented reality technologies, which may produce immersive and interactive rehabilitation experiences.

## 5. Conclusions

This study focuses on the urgent need for inventive interventions in rehabilitation, particularly in light of the difficulties presented by conventional approaches and the increased need for such services in the aftermath of the COVID-19 pandemic. Telerehabilitation is being increasingly adopted as a viable approach to connect patients and healthcare practitioners. It offers benefits such as improved convenience, more accessibility, and the capacity to deliver individualized therapy. The traditional rehabilitation model faces challenges concerning patient motivation and the presence of experienced healthcare practitioners, especially in geographically limited regions, which have been worsened by the recent pandemic. This highlights the need to implement creative interventions, whether under the guidance of healthcare experts or autonomously, as demonstrated by the STASISM platform.

Our research offers insights into the practical consequences of the STASISM platform in technology-driven rehabilitation. It demonstrates the potential of this platform to restructure and optimize healthcare practices, leading to improved patient outcomes and experiences. Conventional rehabilitation encounters ongoing difficulties in inspiring patients and the lack of sufficiently skilled healthcare professionals, particularly in some geographic areas. The combination of telerehabilitation, gamification, and personalization, demonstrated by the adaptable multi-sensor STASISM system, offers a potentially successful solution to these difficulties.

Significantly, the ongoing enhancement of the platform has been greatly influenced by user feedback. The platform’s effective implementation in Ukraine exemplifies its ability to tackle healthcare inequalities in areas marked by a scarcity of healthcare practitioners. The platform’s foundation has been intentionally crafted to be readily accessible, cost-effective, and adaptive to technological progress. The integration of sophisticated sensing technologies and machine learning greatly improves its ability to provide treatment solutions based on empirical evidence.

The framework’s intentional design to ensure accessibility, cost-effectiveness, and adaptability requires continuous research. Subsequent research endeavors should prioritize the improvement of the platform according to user feedback, evaluate its ability to adapt to various healthcare systems and study the long-term effects on patient outcomes to establish its continued effectiveness. Furthermore, it is essential to examine the platform’s influence on healthcare inequalities in different geographical and socioeconomic settings to obtain full understanding.

In the future, research should focus on improving the capabilities of the platform by integrating new sensor technologies and breakthroughs in machine learning. Evaluating the platform’s capacity to adjust to changing technology guarantees its pertinence. Furthermore, it is crucial to investigate the incorporation of STASISM into more comprehensive telehealth initiatives and evaluate its compatibility with current healthcare systems in order to ensure universal acceptance.

To summarize, future research should give priority to continuously enhancing the STASISM platform, investigating its influence on various healthcare environments, and evaluating its long-term efficacy and adaptability. This will enhance comprehension of its capacity to alleviate healthcare disparities and promote favorable patient results.

The STASISM platform is a significant improvement in rehabilitation as it provides technology-driven, personalized, and all-encompassing therapies. These strategies show significant potential in reducing healthcare disparities and enhancing the welfare of patients requiring rehabilitative therapy. In the future, research should focus on continuously improving the platform based on user feedback, examining its ability to work well in different healthcare systems, and evaluating its long-term effectiveness and impact on healthcare inequalities.

## Figures and Tables

**Table 1:** Recommended hardware requirements.

Component	Requirement
OS	Microsoft Windows 10
Memory	8 GB
CPU	64-bit CPU with 4 cores 3+ GHZ
GPU	Nvidia GeForce 950 or equivalent
DirectX	Version 11
Storage	30 GB free
Internet	4G or Broadband
Balance Board	STASISM Balance Board
Webcam	1080 p, 60 fps

**Table 2:** Description of the specific serious games with the controllers and the therapeutic targets.

Games	Description	Mode	Visual Adaptation *	Game Outcome	Controller	Therapeutic Targets	Screenshots
Crazy Racing	Racing game where the player has a task for each level. The character catches the object, races other cars, and explores different roads.	Single	+	Time Completion Percentage (N of bonus coins)	Balance Board: side and forward-backward sways (standing, sitting, kneeling) Camera (body sways)	Balance function, proprioception, visual-spatial ability, navigation skills, attention	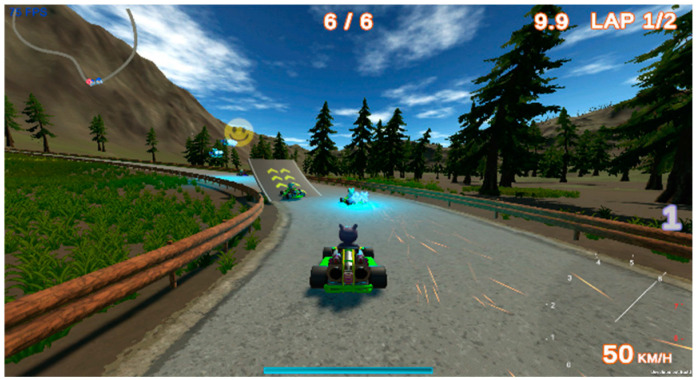
Crusher	Action game that involves different movements of the body parts to crush buildings and transport with a monster character.	Single	−	Points	Camera (upper body, arms, hands, head, mouth)	Motor coordination, proprioception, gross motor skills, fine motor skills, stress relief	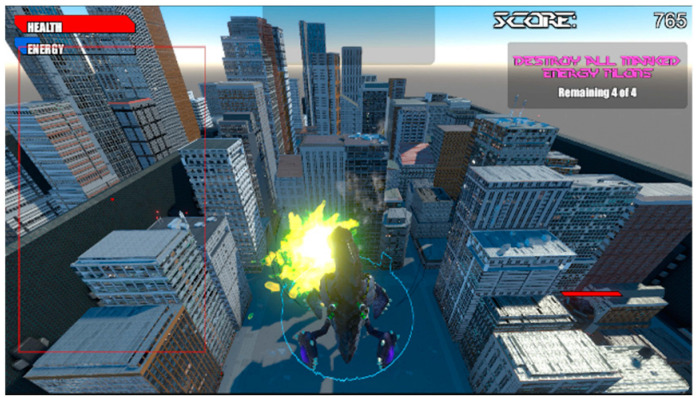
Dodge Dare	Game with augmented reality: a player moves around their room and dodges the lasers	Single	−	Playing Time N of successful dodges	Camera (3D body movement)	Motor coordination, proprioception, visual-spatial ability, gross motor skills, attention	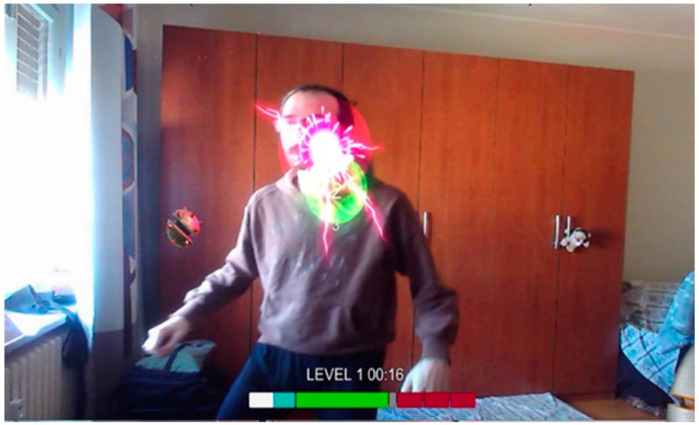
Hex	Game where the player controls a witch flying on her broom with their movements. With each level, the character moves faster, explores different environments, and faces various challenges.	Single	+	Playing Time	Camera (hand clench, mouth)	Gross motor skills, fine motor skills, voluntary motor control, attention	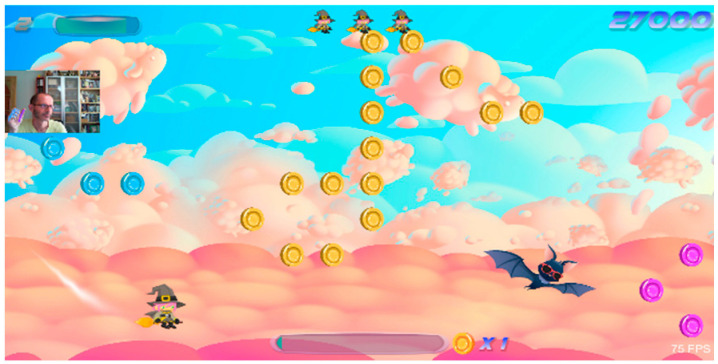
Hungry Woo	Game that revolves around the task of feeding a cat. The main objective is for the player to catch food items while avoiding non-edible objects. As the game progresses, the speed increases and the objects encountered change with each level.	Single, Cooperation, and Player-versus-Player (PvP)	+	Completion Percentage (N of caught edible objects and avoided non-edible objects)	Balance Board: side sways (standing, sitting, kneeling)	Balance function, proprioception, visual-spatial ability, decision-making, object recognition, impulse control	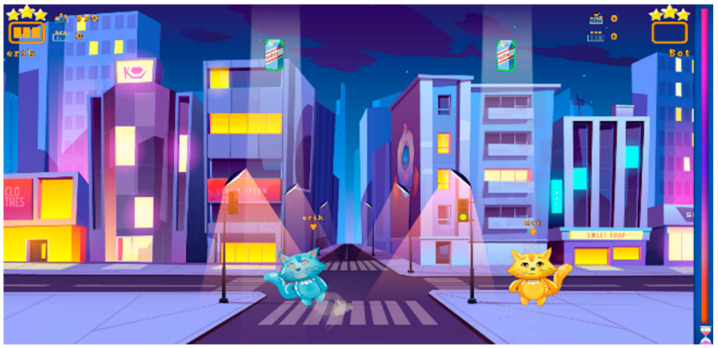
Paddle Waddle	Action game, where the main goal is to keep balls in motion by rebounding them with a paddle. Along the way, players can collect stars to earn points and progress to higher levels. As the child advances, the ball speed increases, and the new puzzle picture opens. In addition to striving for personal achievements, players can compete against others globally to achieve the highest score and receive rewards for surpassing the existing record.	Single	+	Points	Balance Board: side sways (standing, sitting, kneeling) Camera (body sways, shoulder flexion, shoulder abduction, shoulder rotation, elbow flexion, elbow supination, wrist flexion, hand clench, mouth)	Balance function, proprioception, visual-spatial ability, gross motor function, fine motor skills, voluntary motor control, attention, decision-making	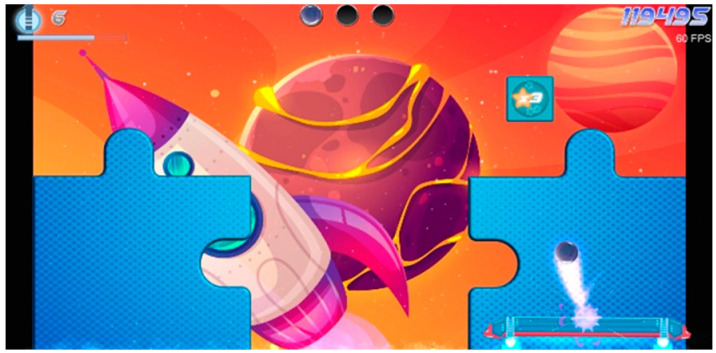
Pongo Paddle	Variation of the favorite Paddle Waddle game. Action game, where the main goal is to keep balls in motion by rebounding them with a paddle. Along the way, players can collect stars to earn points and progress to higher levels. As the child advances, the ball speed increases, and the new puzzle picture opens. In addition to striving for personal achievements, players can compete against others globally to achieve the highest score and receive rewards for surpassing the existing record.	Single	+	Points	Balance Board: forward-backward sways (standing, sitting, kneeling) Camera (body sways, shoulder flexion, shoulder abduction, shoulder rotation, elbow flexion, elbow supination, wrist flexion, hand clench, mouth)	Balance function, proprioception, visual-spatial ability, gross motor function, fine motor skills, voluntary motor control, attention, decision-making	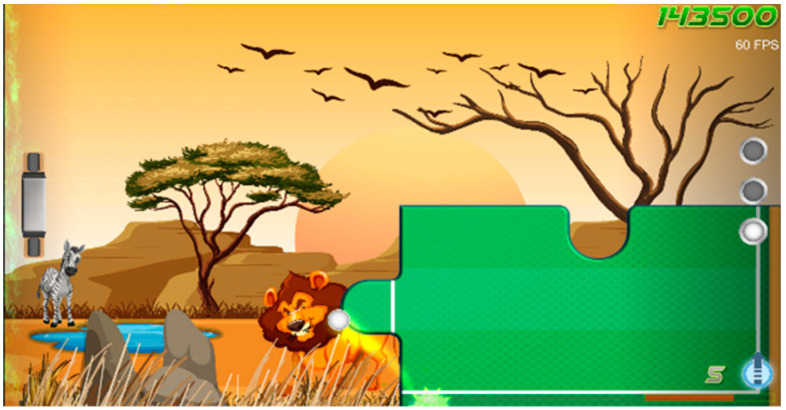
Roxer	A musical game where the player should follow the rhythm to create the melody. It is possible to use the existing song or write their own.	Single	−	Points	Balance Board: side or forward-backward sways (standing, sitting, kneeling) Camera (imitation of the guitar playing)	Balance function, proprioception, visual-spatial ability, gross motor function, fine motor skills, attention	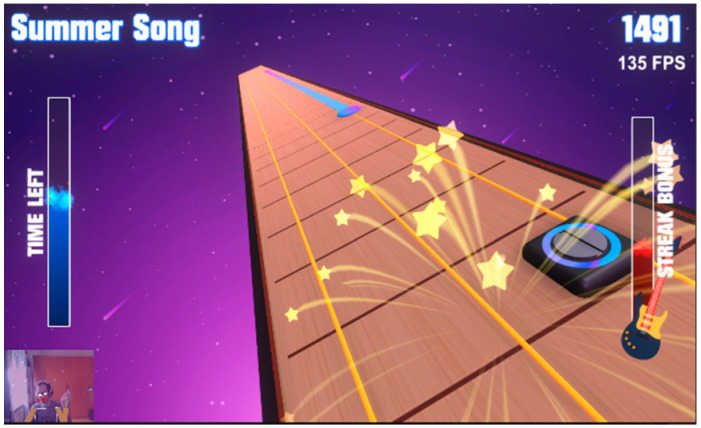
Super Surfer	Super Surfer is a game in which the player gets to control their surfboard and explore new environments when collecting stars, which cumulate and help to earn more points.	Single, Cooperation, and PvP	+	Completion Percentage (N of caught stars)	Balance Board: side and forward-backward sways (standing, sitting, kneeling) Camera (body sways)	Balance function, proprioception, visual-spatial ability, navigation skills, attention	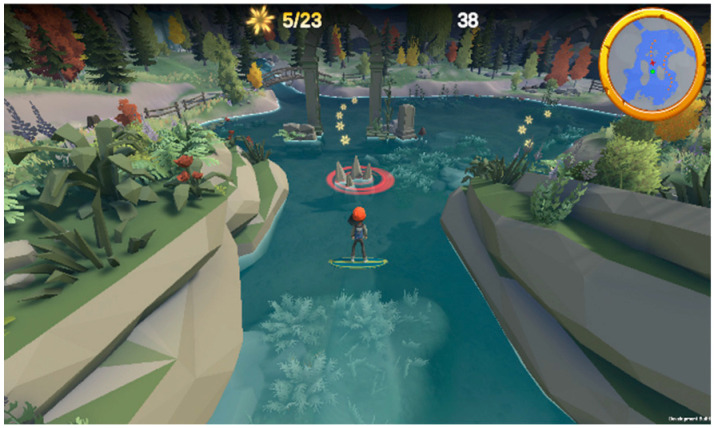
Up You Go	Action game that is meant to be played with both hands and arms. The player controls a character trying to reach the top, shoving falling rocks, disabling moving enemies, and solving puzzles.	Single	−	Points	Camera (imitation of the climbing)	Balance function, proprioception, visual-spatial ability, gross motor skills, fine motor skills, attention	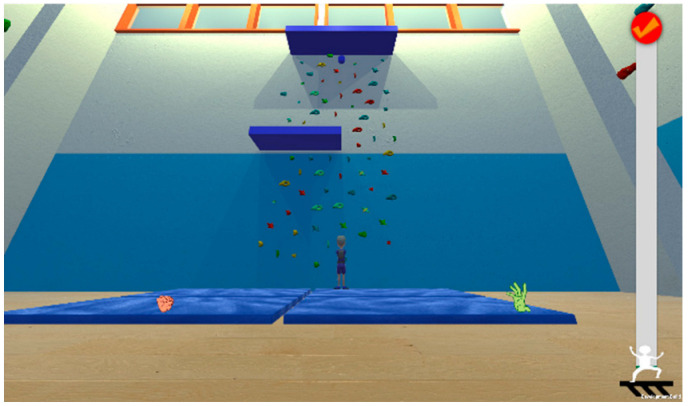
Woo’s Wonderful World Adventure	Puzzle game involving matching falling blocks based on their colors while rescuing caged animals. In the game, players join Woo, a cat who embarks on a global journey in his airplane, visiting various countries. Each country in the game showcases unique artwork inspired by the local culture, creating an immersive and diverse experience. To progress, Woo must solve child-friendly puzzles specific to each country he visits. The game combines puzzle-solving with elements of exploration and cultural appreciation, making it an engaging and educational experience for players.	Single, Cooperation, and PvP	+	Points	Balance Board: side sways (standing, sitting, kneeling)	Balance function, proprioception, visual-spatial ability, attention, decision-making, object recognition, impulse control	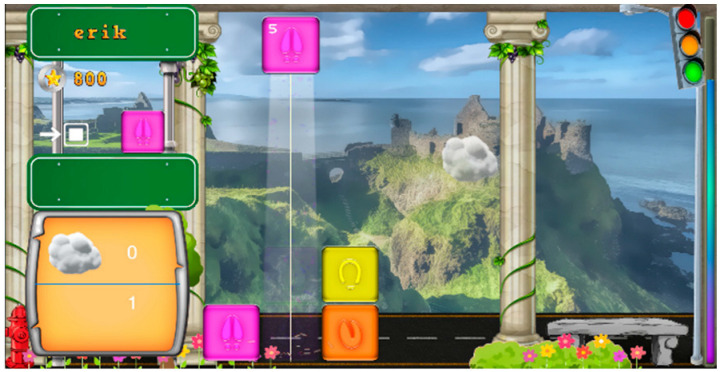

* + denotes the possibility of modify the visual of the games according to various pathologies, − denotes the absence of visual configuration.

**Table 3:** Comparison of the characteristics of different telerehabilitation platforms.

Variable		Platform				
		STASISM	Timocco [44]	EnABLE Games [45]	Jintronix [46]	VAST.Rehab [47]
Therapeutic aim	Gross motor skills	+	−	+	+	+
	Balance	+	+	+	+	+
	Hand function	+	+	+	+	+
	Cognition	+	+	+	+	+
	Participation	+	+	-	+	+
Patients	Children	+	+	+	-	+
	Adults	+	-	+	-	+
	Older adults	−	−	−	+	+
Settings	Home	+	+	+	−	+
	Clinical centers	+	N/A	+	+	+
Controllers		Web camera Nintendo Wii Balance Board	Web camera Two non-specific contrast objects	Kinect Orbbec Astra Leap Motion	Kinect	ZED 2 Stereo Camera Meta Quest 2
Functionality	Serious Games	+	+	+	+	+
	Embedded assessment	+	−	N/A	+	+
	Rehabilitation exercises	+	+	−	+	+
	Virtual Reality	−	−	−	−	+
	Social platform	+	−	−	−	−
Configuration	Analysis of the patient’s data	+	+	+	+	+
	Opportunity to personalize the system	+	+	+	+	+
	Automatic change in exercise difficulty	+	−	−	−	+

## Data Availability

Data are contained within the article.
